# Diagnostic Challenges of Pneumocystis Pneumonia during the COVID-19 Pandemic: A Case of a Young Patient with Ground Glass Opacities and Pulmonary Embolism on Chest CT

**DOI:** 10.1155/2021/5669543

**Published:** 2021-07-31

**Authors:** William Lim, Maham Suhail, Keith Diaz

**Affiliations:** Department of Internal Medicine, Richmond University Medical Center, Staten Island, New York, NY, USA

## Abstract

The coronavirus disease 2019 (COVID-19) pandemic is wreaking havoc across the globe. This pandemic has given rise to a mindset where physicians tend to neglect other causes of pneumonia, especially if the patient presents with signs and symptoms commonly associated with COVID-19. Herein, we report a case of a young man presenting to the emergency department with common clinical, radiological, and laboratory features of COVID-19 pneumonia such as shortness of breath, hypoxia, pulmonary embolism, elevated D-dimer, and bilateral ground glass opacities on computed tomography of the chest but was later diagnosed with *Pneumocystis* pneumonia that was treated with appropriate antibiotics and corticosteroids. This case highlights the importance of performing a thorough clinical history and differentiating the clinical and radiological features of COVID-19 pneumonia from pneumonia of other etiologies.

## 1. Introduction

Since the first case of COVID-19 was diagnosed in December 2019, it has rapidly spread across the globe, resulting in the COVID-19 pandemic. Presently, with more than 100 million cases of COVID-19 globally, it is not uncommon for physicians to think of COVID-19 as an initial diagnosis for patients presenting with pneumonia [1]. In this report, we discuss a case of a young adult with unremarkable past medical history presenting to the emergency department with shortness of breath and diarrhea. Computed tomography (CT) of the chest revealed diffuse pulmonary bilateral ground glass opacities and pulmonary embolism. Recognizing the differences in radiological and clinical features of COVID-19 and *Pneumocystis* pneumonia helped to reach the correct diagnosis and, thus, initiate the appropriate treatment for the patient.

## 2. Case Presentation

A 23-year-old male presented to the emergency department (ED) with a chief complaint of diarrhea for the last one week. On examination, his vital signs were as follows: temperature, 101.8 F; blood pressure, 125/76 mmHg; pulse rate, 126/min; and respiratory rate, 24/min. His blood oxygen saturation was 85% at room air and 95% with 5 L oxygen via nasal cannula. His body mass index (BMI) was 25.1 kg/m^2^. Chest examination revealed decreased breath sounds with bilateral crackles in the lower part of lung fields and normal first and second heart sounds.

Initial laboratory investigations revealed these results: white blood cell count (WBC), 7700/ul with lymphopenia; prothrombin time (PT), 16.6 seconds; international normalized ratio (INR), 1.4; activated partial thromboplastin time (APTT), 38.5 seconds; D-dimer, 3.85 mcg/mL, serum lactate dehydrogenase (LDH), 402 U/L; and C-reactive protein (CRP), 3.63 mg/dl. Arterial blood gas (ABG) analysis showed a pH of 7.53, paCO_2_ of 33 mmHg, paO_2_ of 62 mmHg, serum HCO_3_^−^ level of 27.6 mmol/L, and alveolar-arterial (A-a) oxygen gradient of 46.5 mmHg.

**Figure 1:**
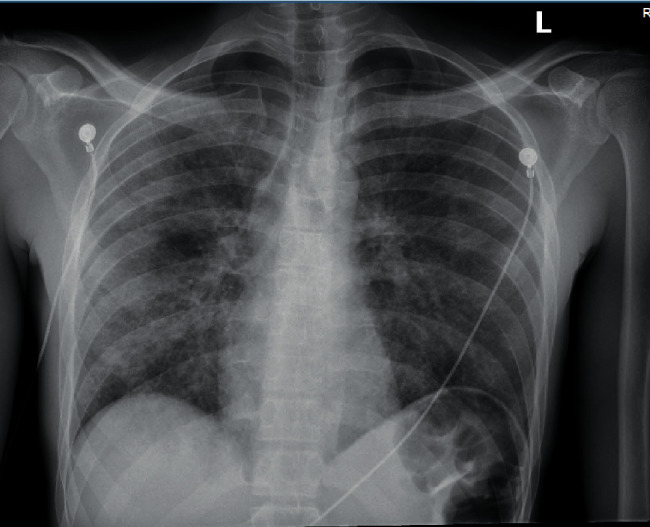
CXR showing bilateral pulmonary infiltrates.

**Figure 2:**
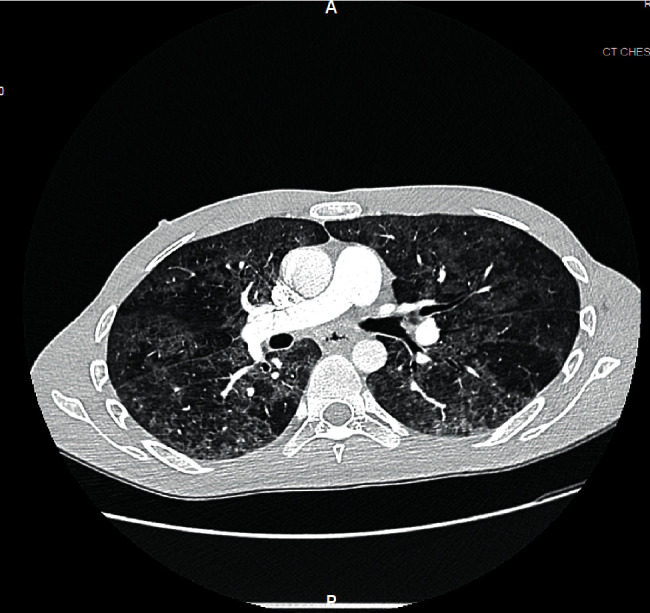
Chest CT showing diffuse bilateral ground glass opacities.

A chest X-ray (Figure 1) showed patchy bilateral pulmonary opacities. The patient's clinical presentation of shortness of breath and hypoxia together with elevated D-dimer levels prompted a chest CT to rule out pulmonary embolism. The chest CT demonstrated notable diffuse bilateral ground glass opacities (Figure 2) and arterial embolism in the pulmonary arteries supplying the right middle and lower lobes.

Reverse-transcription polymerase chain reaction (RT-PCR) for COVID-19 ribonucleic acid (RNA) conducted on a nasopharyngeal swab sample of the patient revealed a negative result. However, the patient was still considered to have clinical COVID-19, given he had typical features of COVID-19 such as hypoxia, shortness of breath, elevated D-dimer, pulmonary embolism, and ground glass opacities on chest CT. Thereby, dexamethasone (6 mg) for COVID-19 and apixaban for pulmonary embolism were initiated. Decision was made to hold remdesivir since COVID-19 was not confirmed in this patient.

**Figure 3:**
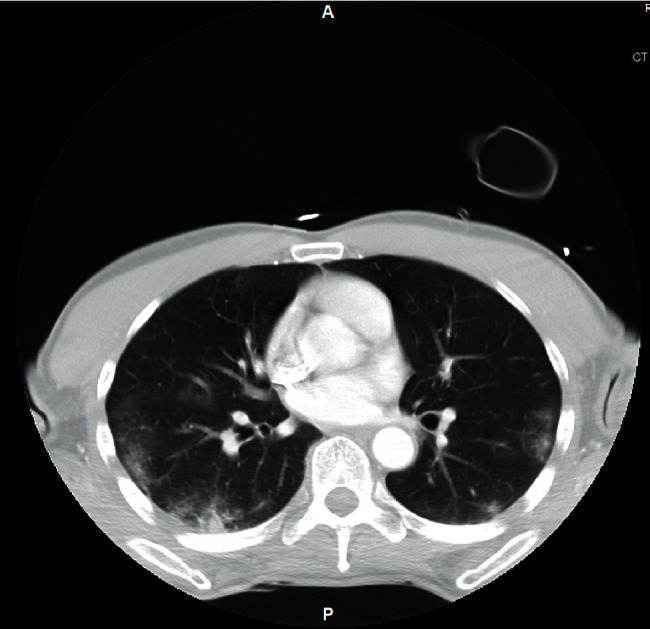
Chest CT of the COVID-19 patient showing patchy and peripheral ground glass opacities mid to lower lobe predominant (taken from another COVID-19-positive patient for comparison).

During further questioning, the patient reported productive yellowish cough during the last month coupled with anorexia and significant weight loss of 15 pounds. The insidious onset of the long-lasting symptoms and the radiological features characterized by ground glass opacities seen in *Pneumocystis* pneumonia as opposed to patchy and peripheral ground glass opacities of typical COVID-19 pneumonia (Figure 3) prompted further investigation for potential occult immunodeficiency. The fourth-generation Ag/Ab combination human immunodeficiency virus (HIV)-1/2 immunoassay showed a positive result for HIV-1 antibody and negative for HIV-2 antibody. Repeat PCR tests for COVID-19 on day 3 and day 5 of hospitalization were negative as well. Thereafter, IV trimethoprim-sulfamethoxazole 400 mg every 8 hours was started for possible *Pneumocystis* pneumonia (PCP) given the new diagnosis of HIV. Dexamethasone was switched to prednisone 40 mg oral twice a day.

Bronchoscopy was performed on day 4 of hospitalization, and bronchoalveolar culture, Gram stain, acid-fast bacilli (AFB) stain, silver stain, and *Pneumocystis jirovecii* PCR were performed on the specimen obtained. Cells obtained via bronchial washing were negative for malignancy, and no fungal elements were visualized by methenamine silver stain. No acid-fast bacilli were detected on AFB staining, and neither any *Mycobacterium* species was isolated in the mycobacterial culture. HIV viral load was 1.4 million copies/ml, and total cluster of differentiation 4 (CD4) count was <20 cells/mcl. Treatment with oral emtricitabine/tenofovir and oral dolutegravir was, thus, initiated.

PCR for *Pneumocystis jirovecii* on material obtained via BAL revealed a positive result. The patient's presentation of chronic symptoms as opposed to acute presentation of COVID-19 patients together with a positive HIV test, high viral load, low CD4 count, and positive PCR for *Pneumocystis jirovecii* contributed to establishing the diagnosis of *Pneumocystis* pneumonia. After one more week of continued treatment with IV antibiotics and prednisone, the patient's condition improved and he was discharged with instructions for home oxygen therapy and prescription for apixaban, trimethoprim-sulfamethoxazole (800 mg/160 mg), prednisone, dolutegravir, and emtricitabine/tenofovir.

## 3. Discussion

In the current situation of the COVID-19 pandemic, there have been an increasing number of patients presenting with severe symptoms of the disease, such as cough, fever, shortness of breath, hypoxia, and diarrhea. *Pneumocystis* pneumonia is also characterized by an insidious onset of similar symptoms when found in association with other opportunistic infections. Given these similarities, the diagnosis of *Pneumocystis* pneumonia may take a backseat in the setting of increasing COVID-19 incidence in the present scenario [2]. In the present case, the patient's presentation of hypoxia, shortness of breath, and diarrhea together with elevated D-dimer and LDH levels along with pulmonary ground glass opacities and pulmonary embolism seen on chest CT made it difficult to differentiate between COVID-19 and *Pneumocystis* pneumonia.

Elevated levels of serum LDH, inflammatory markers (CRP and ferritin), and D-dimer are common laboratory findings associated with COVID-19 [3–5]. Diagnostic tests for COVID-19 include nucleic acid amplification tests (NAAT) such as RT-PCR and antigen testing. RT-PCR assay of COVID-19 RNA from the upper respiratory tract is the preferred initial diagnostic test for COVID-19 with a reported sensitivity of 77% [6, 7].

On the other hand, the common laboratory findings of *Pneumocystis* pneumonia are low CD-4 counts (<200 cells/microliter), widened alveolar-arterial oxygen gradient, and elevated LDH level [8–10]. Diagnostic tests for *Pneumocystis* pneumonia include microscopy with staining and a PCR test. Gomori-methenamine silver is the commonly used stain to detect *Pneumocystis* pneumonia. The sensitivity and specificity of Grocott-Gomori-methenamine silver stain (GMS) are 79.4 and 99.2%, respectively [11]. PCR has a sensitivity of as high as 96% and specificity of more than 90% in detecting *Pneumocystis jirovecii* in respiratory samples [12–14]. PCR is the most sensitive method for the detection of *Pneumocystis* and should be considered the diagnostic test of choice for patients with high suspicion of *Pneumocystis* pneumonia with negative results on GMS stain, as was encountered in the present case [15].

Both COVID-19 and *Pneumocystis* pneumonia can present with ground glass opacities in the lungs on chest CT, and there are no pathognomonic radiographic findings associated with either of the two pathologies that would lead to a definite diagnosis. In general, *Pneumocystis* pneumonia is characterized by diffuse infiltrates mainly involving the upper lobe while sparing the subpleural regions, whereas COVID-19 is characterized by patchy infiltrates, mainly in the lower lobes with peripheral and subpleural distribution [16–18]. COVID-19 is commonly associated with hypercoagulability which can lead to pulmonary embolism [19–21], whereas pulmonary embolism is not a common feature of *Pneumocystis* pneumonia [22]. Elevated LDH is a common laboratory abnormality associated with both COVID-19 and *Pneumocystis* pneumonia, while widened alveolar-arterial oxygen gradient is more commonly found in *Pneumocystis* pneumonia.

Since the treatment protocols of COVID-19 and *Pneumocystis* pneumonia are entirely different, it is very important to reach the correct diagnosis. COVID-19 treatment includes remdesevir, baricitinib, or tocilizumab, while the standard regimen for *Pneumocystis* pneumonia is trimethoprim-sulfamethoxazole for 21 days [23–26]. Corticosteroid of choice used in COVID-19 treatment is dexamethasone with a suggested dosing of 6 mg once daily for 10 days [27–29], while the corticosteroid indicated for moderate-to-severe *Pneumocystis* pneumonia (partial pressure of oxygen <70 mmHg at standard room conditions and/or an A-a oxygen gradient of ≥35 mmHg) is prednisone 40 mg twice a day with subsequent taper [30, 31]. Baricitinib and tocilizumab are immunomodulatory drugs that are indicated for COVID-19 treatment. In the present case, it was of utmost importance to reach the correct diagnosis in order to avoid administration of immunomodulatory agents to an HIV patient with opportunistic infection of *Pneumocystis jirovecii,* which could have led to deterioration in the patient's condition.

## 4. Conclusions

We report a case of *Pneumocystis* pneumonia presenting with features mimicking COVID-19 pneumonia. Increased COVID-19 cases in the scenario of the pandemic have led physicians into neglecting other causes of pneumonia and respiratory failure. This case highlights the importance of medical history taking and detailed clinical examination. It also highlights the importance of understanding the differences between clinical and radiological features of COVID-19 and *Pneumocystis* pneumonia.

## Data Availability

No data were used to support this study.

## Abbreviation

F: Fahrenheit
mmHg: Millimeter of mercury
min: Minute
g/dl: Gram per deciliter
mg/dl: Milligram per deciliter
k/ul: Kilo per microliter
mcg/mL FEU: Microgram/mL fibrinogen equivalent units
U/L: Unit per liter
mmol/L: Millimole per liter
ml: Milliliter
mcl: Microliter.
